# Optimizing Grain Yield and Radiation Use Efficiency through Synergistic Applications of Nitrogen and Potassium Fertilizers in Super Hybrid Rice

**DOI:** 10.3390/plants12152858

**Published:** 2023-08-03

**Authors:** Jun Deng, Jiayu Ye, Xuefen Zhong, Qingqing Yang, Matthew Tom Harrison, Chunhu Wang, Liying Huang, Xiaohai Tian, Ke Liu, Yunbo Zhang

**Affiliations:** 1 MARA Key Laboratory of Sustainable Crop Production in the Middle Reaches of the Yangtze River, College of Agriculture, Yangtze University, Jingzhou 434025, China; 15926599893@163.com (J.D.); 201772394@yangtzeu.edu.cn (C.W.); lyhuang8901@126.com (L.H.); xiaohait@sina.com (X.T.); ke.liu@utas.edu.au (K.L.); 2 Agricultural and Rural Bureau of Duodao District, Jingmen 448000, China; 3 Tasmanian Institute of Agriculture, University of Tasmania, Burnie 7320, Australia; matthew.harrison@utas.edu.au

**Keywords:** nitrogen, potassium, grain yield, biomass production, radiation use efficiency, harvest index, super hybrid rice

## Abstract

The remarkable yield performance of super hybrid rice has played a crucial role in ensuring global food security. However, there is a scarcity of studies investigating the contribution of radiation use efficiency (RUE) to hybrid rice yields under different nitrogen and potassium treatments. In this three-year field experiment, we aimed to evaluate the impact of two hybrid rice varieties (Y-liangyou 900: YLY900 and Quanyouhuazhan: QYHZ) under varying nitrogen regimes (N_90_: 90 kg N ha^−1^, N_120_: 120 kg N ha^−1^, N_180_: 180 kg N ha^−1^) and potassium regimes (K_120_: 120 kg K_2_O ha^−1^, K_160_: 160 kg K_2_O ha^−1^, K_210_: 210 kg K_2_O ha^−1^) on grain yield and its physiological determinants, including RUE, intercepted photosynthetically active radiation (IPAR), aboveground biomass production, and harvest index (HI). Our results revealed that both rice varieties exhibited significantly higher yields when coupled with nitrogen and potassium fertilization. Compared to the N_90_ × K_120_ treatment, the N_120_ × K_160_ and N_180_ × K_210_ combinations resulted in substantial increases in grain yield (12.0% and 21.1%, respectively) and RUE (11.9% and 21.4%, respectively). The YLY900 variety showed notable yield improvement due to enhanced aboveground biomass production resulting from increased IPAR and RUE. In contrast, the QYHZ variety’s aboveground biomass accumulation was primarily influenced by RUE rather than IPAR, resulting in higher RUE and grain yields of 9.2% and 5.3%, respectively, compared to YLY900. Importantly, fertilization led to significant increases in yield, biomass, and RUE, while HI remained relatively constant. Both varieties demonstrated a positive relationship between grain yield and IPAR and RUE. Multiple regression analysis indicated that increasing RUE was the primary driver of yield improvement in hybrid rice varieties. By promoting sustainable agriculture and enhancing fertilizer management, elevating nitrogen and potassium levels from a low base would synergistically enhance rice yield and RUE, emphasizing the critical importance of RUE in hybrid rice productivity compared to HI.

## 1. Introduction

Rice (*Oryza sativa* L.) is one of the most essential food crops worldwide and provides sustenance to over half of China’s population [1]. However, as urbanization and population growth have accelerated, China’s arable land has declined, exacerbating the already strained human–land interaction [2,3]. The country’s ability to maintain food security is at stake due to insufficient per capita arable land resources, compounded by the harmful effects of climate change, natural calamities, land degradation, and environmental pollution [3,4,5]. Thus, increasing rice yield and enhancing sustainable agricultural development are vital to ensure both present and future food security.

In 1996, to enhance rice yield and guarantee food security, China initiated a “super” rice breeding program founded on the ideotype concept. By combining ideotypes and subspecies heterosis, Chinese breeders have created numerous high-yield varieties [6,7]. These newly bred rice varieties have a 12% increase in yield potential compared to ordinary hybrid rice [8,9,10], hence the name “super hybrid rice”. The high potential yield of super rice can be attributed to several factors, such as greater source–sink capacity, higher net photosynthetic rate and leaf area index, greater carbohydrate conversion efficiency, and a higher root biomass and root activity, as well as stronger resistance to lodging [9,11,12]. As of 2023, 133 rice varieties have successfully satisfied the requirements of being designated as super rice varieties in China. Super rice varieties have shown remarkable potential for high yields and have played a significant role in increasing food production in recent decades.

The dry matter accumulation generated by photosynthesis in crops is the primary aspect contributing to crop yield [13,14]. The yield of a crop is contingent on its ability to efficiently utilize light energy. Enhancing the light energy utilization of crops involves two focal points: maximizing the interception of incident light and increasing the efficiency of its conversion into chemical energy [15]. The target of high-yielding crop production, from the aspect of light energy utilization, focuses on the complete utilization of solar energy and converting it efficiently into dry matter accumulation and grains. These targets are commonly evaluated by light energy use, light energy conversion ratio, and radiation use efficiency [16].

Radiation use efficiency (RUE) is the ratio of dry matter accumulation to the intercepted photosynthetically active radiation (IPAR) in the growth stage of crop plants [16]. Monteith further stated that RUE is regulated by three factors: the ability of the canopy to intercept radiation, the efficiency of converting radiation into energy, and the diffusion of CO_2_ into the plant. Leaf photosynthetic rate and N content both impact crop RUE. Accurately calculating RUE is essential for comprehending crop growth and yield [17].

Harvest index (HI), in addition to biomass, is another vital factor influencing rice yield, and it is possible to enhance yield by increasing one or both factors [18]. Traditionally, rice varieties were developed by improving the harvest index for yield enhancement. However, modern rice varieties have been optimized through increased biomass accumulation. Yield can be computed as the product of intercepted radiation, RUE, and HI while ignoring the contribution of photosynthesis to root growth [19]. Accordingly, a simultaneous increase in both intercepted radiation and RUE may potentially elevate the yield further.

Super rice varieties require large inputs of nitrogen fertilizer to achieve their high yield potential, primarily relying on high biomass accumulation [10,20]. Nitrogen fertilizer plays a critical role in determining biomass by regulating IPAR and RUE [21,22]. Additionally, the nitrogen content per unit area of the leaf also affects radiation use efficiency [17]. Nitrogen application is critical for crop production, as it determines potential biomass production by affecting PAR and RUE [21,22]. Katsura et al. (2007) attributed the high yields of Liangyoupeijiu (LYPJ) to its large leaf area and duration, resulting in significant biomass accumulation under high nitrogen conditions, rather than its RUE [11]. Higher nitrogen rates in hybrid rice varieties result in higher yield potentials due to increased radiation use efficiency (RUE) and aboveground biomass [23,24]. While crop RUE cannot fully explain the yield superiority of ‘‘super’’ hybrid rice [9], a previous study suggested that RUE plays a more prominent role than HI in yield potential in super hybrid rice [19]. Reduced nitrogen fertilization coupled with higher plant density can lead to early canopy closure, higher RUE, and ultimately, increased biomass production [25].

Most research on radiation use efficiency has focused on factors such as nitrogen fertilization [9,10], varieties [9,19], density [25], and light regulation [26]. However, few studies have explored the impact of nitrogen–potassium interaction on radiation use efficiency in super rice, or how intercepted radiation and RUE contribute to super hybrid rice yield. To rectify this, we conducted a three-year field experiment using two hybrid rice varieties with the aims of (1) comparing differences in grain yield, biomass production, intercepted radiation, and RUE of the two hybrid rice varieties under different N and K rates, and (2) identifying the relationships between grain yield and intercepted radiation or RUE in super hybrid rice under different N and K treatments.

## 2. Results

### 2.1. Weather Conditions

**Figure 1:**
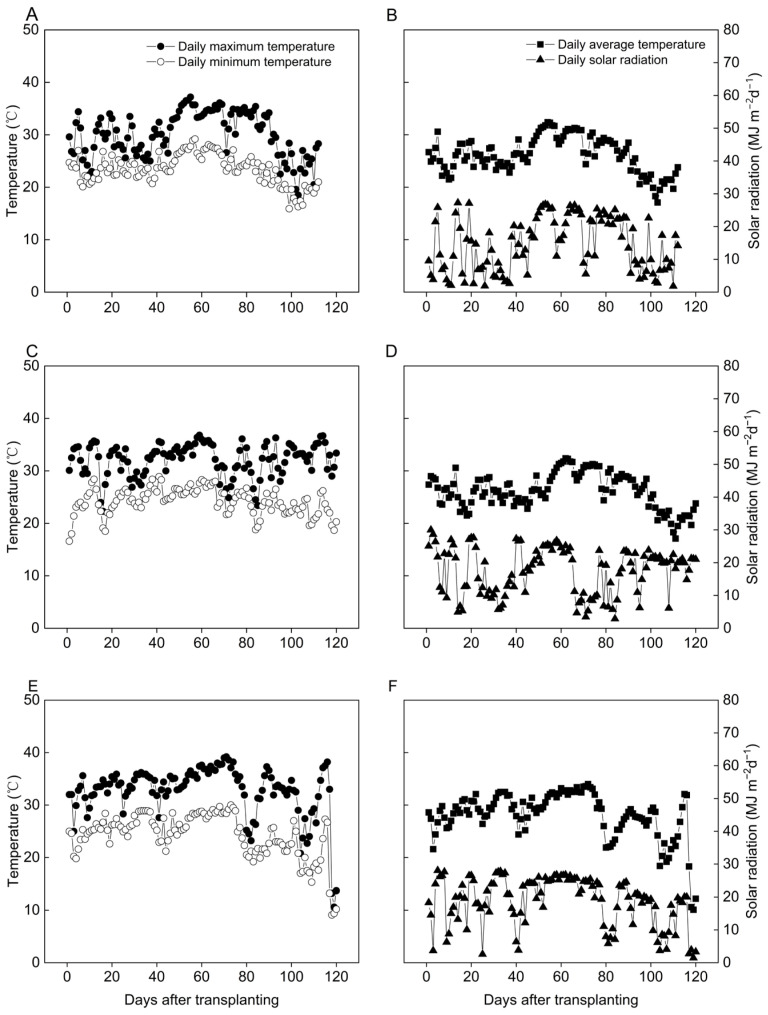
Daily maximum temperature, minimum temperature, average temperature, and solar radiation from transplanting to maturity in 2020 (**A**,**B**), 2021 (**C**,**D**), and 2022 (**E**,**F**) in Jingzhou, Hubei Province, China.

The daily maximum temperature increased from 37.2 °C in 2020 to 36.8 °C in 2021 and significantly rose to 39.2 °C in 2022 (Figure 1). Similarly, the corresponding values for minimum temperature were measured as 15.9 °C and 16.6 °C in 2020 and 2021, while in 2022, it decreased to 15.3 °C. Furthermore, the total solar radiation received during the rice growth periods varied across the three years, with recorded values of 1622.9 MJ m^−2^, 2091.9 MJ m^−2^, and 2180.9 MJ m^−2^ in 2020, 2021, and 2022, respectively.

Although there was no significant variance in the temperature range between 2020 and 2021, the maximum temperature in 2022 was significantly higher. Additionally, while the total solar radiation received in 2021 and 2022 showed no significant difference, it notably increased by 28.9% and 34.4%, respectively, compared to that of 2020.

### 2.2. Grain Yield

**Figure 2:**
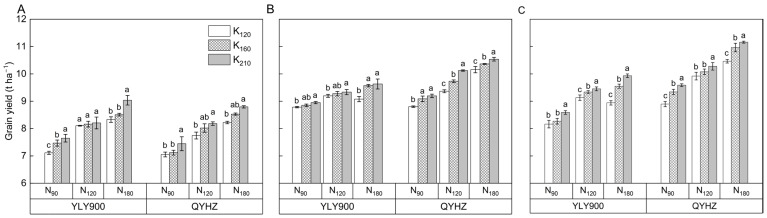
Grain yield of two hybrid rice varieties under three nitrogen and three potassium application rates in 2020 (**A**), 2021 (**B**), and 2022 (**C**). Vertical bars indicate standard errors (*n* = 3). Means followed by the same letter are not statistically different (LSD, *p* < 0.05).

The utilization of N and K had a significant impact on the grain yield over the three-year period of the study (Figure 2). A notable increase in grain yield of the two hybrid rice varieties was observed upon increasing N application, with an average surge of 8.8% in the N_120_ treatment and 14.2% in the N_180_ treatment compared to that of the N_90_ treatment. The average grain yield was also higher under the K_160_ and K_210_ treatments than under the K_120_ treatment, with increases of 3.0% and 5.5%, respectively. Moreover, the two rice varieties exhibited significantly different yields, with QYHZ exhibiting a 5.3% higher yield than YLY900. Compared to N_90_ × K_120_ treatment, the grain yields of N_120_ × K_160_ and N_180_ × K_210_ were increased by 11.9% and 21.1%, respectively.

### 2.3. Aboveground Total Dry Weight and HI

**Figure 3:**
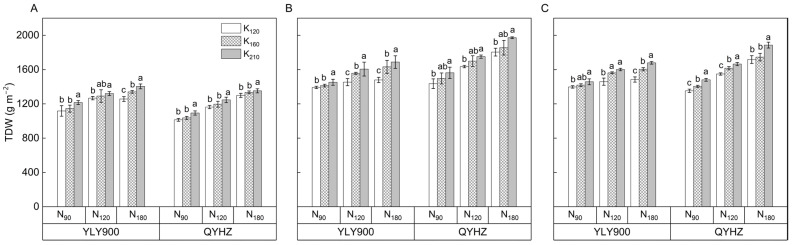
The aboveground total dry weight (TDW) of two hybrid rice varieties at maturity in 2020 (**A**), 2021 (**B**), and 2022 (**C**). Vertical bars indicate standard errors (*n* = 3). Means followed by the same letter are not statistically different (LSD, *p* < 0.05).

The application of N and K, as well as their interactions, significantly affected the aboveground total dry weight (TDW) of different rice varieties (Figure 3). Specifically, the three-year average TDW increased by 11.5% and 19.4% in the N_120_ and N_180_ treatments, respectively, as compared to the N_90_ treatment.

The application of K fertilizer also significantly increased the TDW of rice, with a respective increase of 4.2% and 8.5% in the K_160_ and K_210_ treatments compared to the K_120_ treatment. Concerning the varieties, the average TDW of QYHZ was 4.3% higher than that of YLY900. YLY900 and QYHZ had the highest TDW under the N_180_ × K_210_ treatment, with respective increases of 22.1% and 37.0% compared to the N_90_ × K_120_ treatment.

**Figure 4:**
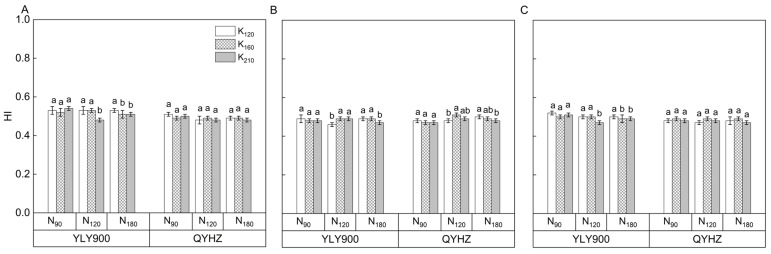
The harvest index (HI) of two hybrid rice varieties at maturity in 2020 (**A**), 2021 (**B**), and 2022 (**C**). Vertical bars indicate standard errors (*n* = 3). Means followed by the same letter are not statistically different (LSD, *p* < 0.05).

The application of N had no significant effect on the HI of the two rice varieties, whereas potassium showed a trend of increasing HI (Figure 4). The interaction between nitrogen and potassium had a significant effect on the HI. Concerning the varieties, the average HI of YLY900 was 3.2% higher than that of QYHZ over the three years.

### 2.4. Growth Durations, IP, IPAR, and RUE

The application of N and K, as well as their interactions, had an extremely significant effect on IP, IPAR, and RUE, with the interaction effect being significant on RUE (Table 1, Table 2, Table 3 and Table 4).

The application of N and K both significantly increased IPAR for the two varieties. Specifically, compared to the N_90_ treatment, the three-year average IPAR increased by 2.8% and 6.4% in the N_120_ and N_180_ treatments, respectively. The increase in IPAR ranged from 1.5% to 3.4% with an increase in potassium. Regarding varieties, YLY900 had an overall 6.0% higher three-year average IPAR than QYHZ.

As with IPAR, the application of N and K significantly increased the light interception percentage (IP). Specifically, compared to the N_90_ treatment, the three-year average IP increased by 2.0% and 5.0% in the N_120_ and N_180_ treatments, respectively, and by 0.8% and 2.1% in the K_160_ and K_210_ treatments, respectively, compared to the K_120_ treatment. Although the differences between the two varieties were small, QYHZ had a slightly higher IP than YLY900.

The two varieties showed significant differences in RUE under different N and K treatments. An increase in N application resulted in an increase in RUE ranging from 8.8% to 12.8%, while an increase in K application resulted in an increase in RUE ranging from 2.5% to 4.7%. The differences between the two varieties were significant, with QYHZ having an overall 9.2% higher RUE than YLY900.

Regarding the interaction effect of nitrogen and potassium, compared to the N_90_ × K_120_ treatment, the three-year average RUE increased by 21.4% in the N_180_ × K_210_ treatment.

The growth duration of the two rice varieties differs significantly, being on average 7–8 d longer for YLY900 compared to QYHZ. There is a trend of nitrogen fertilization prolonging rice growth duration, while potassium fertilization does not have a significant impact on rice growth duration.

### 2.5. Relationships between Grain Yield and TDW or HI

**Figure 5:**
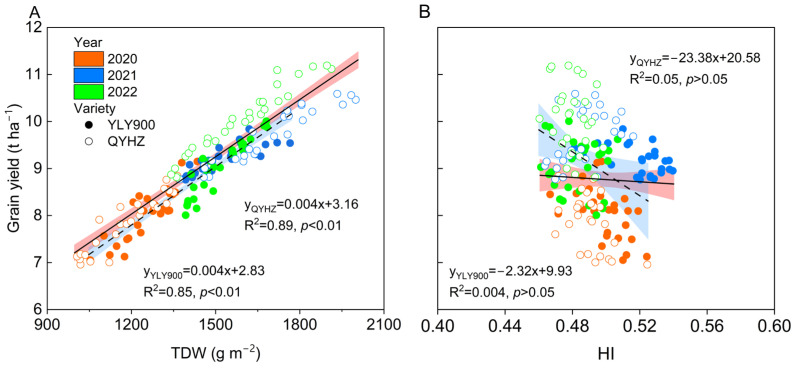
The relationship between total dry weight (TDW) (**A**), harvest index (HI) (**B**), and grain yield from 2020 to 2022. Different colors represent different years; solid circles represent YLY900, hollow circles represent QYHZ; shaded areas represent 95% confidence intervals.

In addition to HI, the TDW in both rice varieties had an extremely significant correlation with yield (*p* < 0.01) (Figure 5). Specifically, QYHZ and YLY900 had the highest correlation coefficient with R^2^ values of 0.89 and 0.85 for TDW, respectively.

### 2.6. Relationships between Grain IR, IP, IPAR, RUE, and Yield

**Figure 6:**
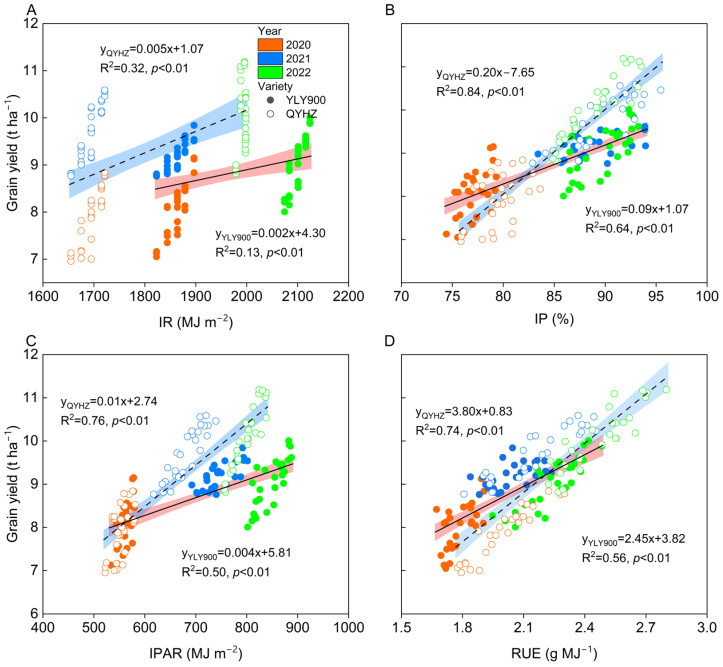
The relationship between total solar radiation (IR) (**A**), light interception percentage (IP) (**B**), intercepted photosynthetically active radiation (IPAR) (**C**), radiation use efficiency (RUE) (**D**), and grain yield of two different varieties was examined across three different rates of nitrogen and potassium application from 2020 to 2022. Different colors represent different years; solid circles represent YLY900, hollow circles represent QYHZ; shaded areas represent 95% confidence intervals.

The IR, IP, IPAR, and RUE of the two rice varieties also had extremely significant correlations with yield (*p* < 0.01) (Figure 6). Specifically, QYHZ had the highest correlation coefficients with R^2^ values of 0.84 and 0.76 for IP and IPAR, respectively. YLY900 had the highest correlation coefficients of 0.64 and 0.56 for IP and RUE, respectively.

### 2.7. Evaluation of the Effects of IPAR, RUE, and HI on Grain Yield in Each Cultivar

The following multiple regression models were developed to quantify the relative contributions of IPAR, RUE, and HI to grain yield (Table 5).

Ln (Grain yield _QYHZ_) = 2.95 + 0.004 ln (IPAR) +3.57 ln (RUE) + 2.71 ln (HI)

Ln (Grain yield _YLY900_) = −3.68 + 0.001 ln (IPAR) + 4.15 ln (RUE) + 2.93 ln (HI)

The multiple regression analysis of yield with IPAR, RUE, and HI for the two rice varieties showed that IPAR had an extremely significant impact on yield for both QYHZ and YLY900 (*p* < 0.001), with regression coefficients of 0.004 and 0.001, respectively. Additionally, RUE had an extremely significant impact on yield for both varieties, with regression coefficients of 3.57 and 4.15. Furthermore, the R^2^ for yield with IPAR, RUE, and HI was 0.90 for QYHZ and 0.70 for YLY900, respectively.

## 3. Discussion

The breakthrough in super hybrid rice yield relies heavily on investment in fertilizers, with nitrogen and potassium fertilizers being the two most essential nutrients in rice production. Research suggests that increased application of nitrogen fertilizer can significantly improve the yield potential of super hybrid rice, which is more likely to achieve high yields when subjected to high nitrogen treatments [9,10,27]. Moreover, studies indicate that rice yield increases with the amount of nitrogen fertilizer applied, but the increase in productivity gradually declines as the amount of nitrogen fertilizer applied is further raised [28,29]. A study that compared the effects of potassium fertilizer on yield for multiple rice varieties demonstrated that the application of potassium fertilizer can significantly increase rice yield [29]. The addition of potassium fertilizer could also substantially enhance rice stem strength, helping to reduce the lodging index of rice, thereby avoiding a decline in yield due to fallen rice plants. Nitrogen fertilizer usage has the most significant impact on rice yield, with potassium fertilizer usage having a relatively minor effect [30]. However, applying nitrogen and potassium fertilizers together, in addition to a supply of micronutrients, can increase rice production even further [31].

The results of a three-year trial indicate that increased application of both nitrogen and potassium fertilizers can further improve rice yield compared to low nitrogen and low potassium treatments. When compared to the N_90_ treatment, the average yield of the two rice varieties under study increased by 11.5% under the N_120_ and N_180_ treatments. Furthermore, compared to the K_120_ treatment, the application of additional potassium fertilizer increased rice yield by 4.3%. Compared to the low nitrogen and low potassium (N_90_ × K_120_) treatment, the rice yield under the N_120_ × K_160_ treatment and N_180_ × K_210_ treatment improved by 11.9% and 21.1%, respectively. These findings indicate that a substantial increase in rice yield can be achieved by further increasing the application of nitrogen and potassium fertilizers based on low nitrogen and low potassium treatments, consistent with previous research results [30,31,32,33,34].

As most high-yielding rice varieties have already achieved optimal plant, their harvest index tends to remain stable, which means further increasing rice yield relies mainly on enhancing biomass accumulation. Numerous studies have demonstrated that increased nitrogen fertilizer can significantly improve rice dry matter accumulation [9,10,34]. Moreover, potassium also plays a crucial role in increasing rice yield. Research suggests that applying potassium fertilizer can enhance chlorophyll content and the proportion of photosynthetic pigments in rice, thereby increasing photosynthetic efficiency and the accumulation of photosynthetic products [35]. In this study, further increasing nitrogen fertilizer application under low nitrogen conditions led to a 15.5% increase in dry matter accumulation, while augmenting potassium fertilizer application under low potassium conditions produced a 6.4% increase in dry matter accumulation. Additionally, compared to the low nitrogen and low potassium (N_90_ × K_120_) treatment, the dry matter accumulation for rice increased by 15.7% and 29.5% under the N_120_ × K_160_ treatment and N_180_ × K_210_ treatment, respectively. Moreover, in this study, increased application of nitrogen and potassium fertilizers could enhance the harvest index of the two rice varieties under study, yet, the extent of the improvement was minor. Therefore, it is advantageous to increase nitrogen and potassium fertilizer application under low nitrogen and low potassium conditions to enhance dry matter accumulation. The increase in yield mainly stems from the elevation in biomass accumulation, consistent with previous research findings [9,19].

RUE is one of the three important resource use efficiencies in crops and is an essential indicator for measuring crop yield [36]. Our results show that both IPAR and RUE contribute to an increase in rice yield, but the physiological mechanisms responsible for yield improvement varied among different varieties. Previous studies showed that Y-liangyou 900 has a yield advantage due to factors such as higher chlorophyll concentration, larger leaf area, and higher photosynthetic and biomass accumulation during the grain-filling stage [26,37]. It also has a longer grain-filling duration, a higher harvest index, and better nitrogen and potassium use efficiency compared to other rice varieties [34]. These traits make Y-liangyou 900 a promising rice variety for improving production. In our study, increased application of nitrogen and potassium fertilizers can improve grain yield and radiation use efficiency. Analysis of the causes revealed that YLY 900 has a long growth duration, leading to a substantial increase in IPAR and biomass, thus improving rice yield and radiation use efficiency [38,39]. Y-liangyou 900’s higher RUE is likely due to its superior leaf photosynthetic performance or improved leaf photosynthetic traits [23]. It has been confirmed in previous studies that Y-liangyou 900 has a higher leaf photosynthetic rate than Liangyoupeijiu, especially during later growth stages [26,28].

QYHZ is a new indica-type three-line hybrid rice variety selected in 2017. The higher IPAR in QYHZ was not caused by its longer growth duration because QYHZ did not have longer growth durations than YLY900 in this study. Instead, the higher IPAR in QYHZ was due to higher IP. Our analysis showed that the yield advantages in QYHZ are primarily due to higher aboveground biomass production resulting from higher RUE, rather than from IPAR. Compared to YLY900, the shorter growth durations of QYHZ resulted in lower IPAR (6.0%) but higher biomass (4.3%), RUE (9.2%), and yield (5.3%). Considering the yield advantages of QYHZ and YLY900, and the results of the multiple regression analyses (Table 5), we conclude that RUE plays a more important role in yield improvement in hybrid rice varieties than HI, which is consistent with our previous studies [19]. Additionally, further combined application of nitrogen and potassium fertilizers under low nitrogen and low potassium (N_90_ × K_120_) conditions can significantly improve radiation use efficiency, thus increasing dry matter accumulation and yield of rice. This suggests that increasing the amount of nitrogen and potassium fertilizer applied in rice production can help to improve the dry matter conversion efficiency of rice, thus increasing rice yield.

Rice yields in China’s superior high-yielding breeding programs have increased significantly due to genetic crop improvements and better crop management practices. Developing a larger or heavier panicle is a crucial method for crop breeders to increase rice yield potential [7,9,19]. Crop physiologists are also moving towards a multi-panicle approach to improve yields, which coincides with their efforts to increase RUE. Our study results can benefit both breeders and agronomists by providing insights on adjusting breeding targets, methodologies, and management practices for the development of super hybrid rice varieties.

However, our study also has some limitations. The contributions of IPAR and RUE to aboveground biomass accumulation and yield under different N and K treatments were only assessed in one ecological region. Solar radiation varies with latitude, altitude, weather conditions, and sunshine hours, which can affect crop RUE and IPAR. Previous studies showed that the RUE of Liangyoupeijiu varied greatly in different ecological regions [11,39]. It is uncertain whether such differences would affect the contributions of IPAR and RUE to aboveground biomass accumulation in these rice varieties under different N and K rates. Therefore, further research is needed to confirm these contributions of super hybrid rice across multiple ecological regions. In addition, the lack of no N and no K treatments in this study is somewhat of a flaw when assessing the independent and reciprocal effects of N and K. Therefore, there is a need to design more reasonable field experiments and multi-ecological area joint experiments to further validate our results.

## 4. Materials and Methods

### 4.1. Experimental Site and Test Material

From 2020 to 2022, a three-year field study was conducted at Yangtze University’s experimental farm in Jingzhou, Hubei Province, China (112°31′ E, 30°21′ N). Prior to each year’s experiment, soil samples from the top 20 cm were gathered to assess soil qualities. Soil samples were collected from the four corners and the center of each treatment plot. The yearly soil characteristic value was then calculated using the average of the four soil samples. The soil in this area is an alluvial calcareous soil, with an average pH of 6.7, 19.7 g kg^−1^ of organic matter, 1.97 g kg^−1^ of total nitrogen, 28.6 mg kg^−1^ of readily available phosphorus, and 126.7 mg kg^−1^ of available potassium over the three-year period. The daily average temperatures during the rice growing seasons in 2020, 2021, and 2022 were 26.2 °C, 26.2 °C, and 28.5 °C, respectively (Figure 1). The average temperature in 2022 was significantly higher than that of 2020 and 2021.

Y-liangyou 900 (YLY900) and Quanyouhuazhan (QYHZ) were used as test materials in the experiment. YLY900 is a new elite super hybrid rice variety, and it was developed using Y58S as the parent and Huazhan as the father. The other testing material, QYHZ, is a new three-line hybrid rice variety of the indica type. It was selected using Quan 9311A as the parent and Huazhan as the father.

### 4.2. Experimental Design and Crop Management

Treatments were arranged in a split–split plot design with three N treatments [N_90_ (90 kg N ha^−1^), N_120_ (120 kg N ha^−1^), and N_180_ (180 kg N ha^−1^)] as the main plots, and three K treatments [K_120_ (120 kg K_2_O ha^−1^), K_160_ (160 kg K_2_O ha^−1^), and K_210_ (210 kg K_2_O ha^−1^)] and varieties (YLY900: Y-liangyou 900 and QYHZ: Quanyouhuazhan) as subplots. The study was conducted with three replicates for each year, with each plot measuring 30 m^2^. Urea was applied at 50%, 30%, and 20% during the transplanting, middle tillering (MT), and panicle initiation (PI) phases, respectively. At PI, split K fertilizer treatments in the form of KCl were used, with 50% applied as a basal dressing and the remaining 50% broadcast. In addition, 100 kg P_2_O_5_ ha^−1^ was applied as a basal fertilizer in the form of calcium superphosphate.

Pre-germinated seeds were planted at a rate of 25 g m^−2^ in a seedbed, and seedlings were transplanted to field plots at 27- to 31-days old, with hill spacings of 20 cm × 24 cm and two seedlings per hill. The management of crops adhered to accepted cultural practices. Transplantation dates were 12 June 2020, 4 June 2021, and 10 June 2022, which are typical timings for planting in Jingzhou. Pesticides were extensively used to control insects and prevent yield and biomass losses.

### 4.3. Sampling and Measurements

Lu’s method [40] was employed to determine the soil properties. Specifically, the pH value was determined through the potentiometric method, the soil organic matter content was determined using the potassium dichromate volumetric method, alkali-hydrolyzable nitrogen was determined employing the alkali hydrolysis diffusion method, soil available potassium was measured using the flame photometric method, and available phosphorus was assessed using the sodium hydrogen carbonate solution-Mo-Sb anti spectrophotometric method.

During the MT, PI, heading (HD), and maturity (MA) stages, a total of six hills of plants were collected and sampled. These plant samples were then separated into straws, leaves, and panicles (only at the HD and MA stages). The leaves from each treatment were flattened out upon a whiteboard (with a 1 cm scale for reference), and later oven-dried at 70 °C until a constant weight was reached to determine the dry weight. Meanwhile, the straw, leaves, and panicles were also oven-dried at 70 °C to determine the dry weight and estimate the total dry weight of the aboveground section (TDW) and yield components at the MA stage through the diagonally sampled plants from six hills in a 5 m^2^ harvest area. The number of panicles per hill was also calculated to obtain the number of panicles per square meter, with the plants being divided into panicles, leaves, and straws. The hand-threshed panicles were separated from unfilled spikelets by immersing them in tap water while three 30 g subsamples of filled spikelets and three 3 g subsamples of unfilled spikelets were examined to determine the spikelet count. After oven-drying to reach a constant weight at 70 °C, the dry weights of the rachis along with the filled and unfilled spikelets were determined. The total dry weight of the straw, rachis, and filled and unfilled spikelets was utilized in the calculation of the aboveground TDW. The spikelets per panicle and grain filling percentage (100 filled spikelet number/total spikelet number) were obtained. Finally, the grain yield was computed and adjusted to a moisture level of 0.14 g H_2_O g^−1^, all of which were conducted based on a 5 m^2^ plot area.

### 4.4. Measurement of IR and RUE

The SunScan Canopy Analysis System, manufactured by Delta-T Devices Ltd. located in Burwell, Cambridge, UK, was utilized to measure light interception by the canopy of the crops at the MT, PI, HD, and MA stages between 11.00 and 13.00 h. For each plot, the canopy’s light intensity was gauged by placing the light bar midway between two rows, just above the water’s surface. Three readings were taken between rows, and another three readings were also captured within rows. Simultaneously, the quantity of incident light was measured. The percentage of intercepted light intensity by the canopy was calculated as [100 × (incoming light intensity − light intensity inside the canopy)/incoming light intensity]. IPAR was estimated to account for 0.45% of the overall solar radiation [41].

The average canopy light absorption and the overall solar radiation absorbed throughout the development phase were utilized to estimate the IPAR during each development stage. Specifically, this was calculated via [1/2(beginning of the development stage canopy light interception and ending of the growth stage canopy light interception) cumulative radiation exposure during the growth phase]. The intercepted radiation from each growth phase was aggregated to determine the IPAR for the entire growing season. Meanwhile, the TDW-to-IPAR ratio of the aboveground section for the complete growing season was used in the computation of RUE. To measure the minimum and maximum temperatures and sun radiation daily, a Vantage Pro2 weather station made by Davis Instruments Corp. (Hayward, CA, USA) was employed.

### 4.5. Quantifying the Contributions of IPAR, RUE, and HI to Grain Yield

The harvest of crops can be calculated as the multiplication of two major factors, namely biomass and harvest index (HI), whereas biomass production is the outcome of radiation usage efficiency (RUE) and the amount of radiation absorbed by the crop canopy. Assuming that the effect of photosynthesis on root growth is disregarded, the crop yield can be expressed as:

Grain yield = IPAR × RUE × HI(1)

In this study, to evaluate and contrast the relative impacts of IPAR, RUE, and HI on the production of grain yield for the two hybrid rice varieties, the following equation was employed:

Ln (Grain yield) = a + b ln (IPAR) + c ln (RUE) + d ln (HI)(2)

The distinctions in yield among hybrids can be explained through the values of coefficients a, b, c, and d assigned to each hybrid. The standard errors of these coefficients’ estimates signify the variability that exists between the hybrids [19].

### 4.6. Data Analysis

Data were analyzed using a three-way analysis of variance with Statistic 8.0 (Analytical Software, Tallahassee, FL, USA). The means were compared between N treatments, K treatments, and varieties based on the least significant difference (LSD) test at a 0.05 probability level.

## 5. Conclusions

In this experiment, we investigated the effects of varying rates of nitrogen and potassium fertilization on the productivity of hybrid rice, including yield, biomass, harvest index, and radiation use efficiency. We also assessed the individual contributions of intercepted photosynthetically active radiation, radiation use efficiency, and harvest index to hybrid rice grain yield. Our findings indicate that increasing the application rates of nitrogen and potassium fertilizers from a low base has a mutually beneficial effect on both biomass production and radiation use efficiency, resulting in an overall increase in rice yield. Importantly, our analysis indicated that radiation use efficiency plays a more crucial role in driving hybrid rice yield than intercepted photosynthetically active radiation and harvest index. These findings highlight the importance of carefully managing nitrogen and potassium fertilizer application to maximize productivity. Furthermore, our study emphasizes the significance of radiation use efficiency in supporting yield, calling for further research to explore strategies for optimizing this critical aspect of hybrid rice production.

## Figures and Tables

**Table 1:** Radiation use efficiency (RUE) and its related parameters of two hybrid rice varieties at maturity under different nitrogen rates in 2020.

Variety	Nitrogen	Potassium	GD	IR	IP	IPAR	RUE
(V)	(N)	(K)	(d)	(MJ m^−2^)	(%)	(MJ m^−2^)	(g m^−2^)
YLY900	N_90_	K_120_	109	1591.4	75.4 b	539.7 c	2.07 b
		K_160_	109	1608.7	76.0 b	550.4 b	2.08 b
		K_210_	109	1622.9	77.7 a	567.2 a	2.14 a
		Mean	109	1607.7	76.4B	552.4 B	2.10 B
	N_120_	K_120_	110	1608.7	75.8 b	548.7 c	2.31 a
		K_160_	110	1622.9	77.0 b	562.1 b	2.30 a
		K_210_	110	1623.9	79.0 a	577.2 a	2.29 a
		Mean	110	1618.5	77.3 AB	562.7 A	2.3 A
	N_180_	K_120_	111	1622.9	77.2 b	563.9 b	2.23 c
		K_160_	111	1623.9	77.3 b	565.2 b	2.37 b
		K_210_	111	1625.4	78.9 a	577.2 a	2.43 a
		Mean	111	1624.1	77.8 A	568.8 A	2.34 A
QYHZ	N_90_	K_120_	102	1529.9	77.5 a	533.8 a	1.90 b
		K_160_	102	1532.7	78.0 a	538.1 a	1.93 b
		K_210_	102	1539.3	79.9 a	553.4 a	1.98 a
		Mean	102	1534.0	78.5 A	541.8 B	1.94 C
	N_120_	K_120_	102	1532.7	79.4 a	547.9 a	2.12 b
		K_160_	102	1539.3	79.6 a	551.7 a	2.17 ab
		K_210_	102	1556.7	80.2 a	561.6 a	2.22 a
		Mean	102	1542.9	79.8 A	553.7 AB	2.17 B
	N_180_	K_120_	104	1539.3	79.7 a	551.8 a	2.36 a
		K_160_	104	1556.7	80.6 a	564.3 a	2.37 a
		K_210_	104	1563.4	80.8 a	568.3 a	2.38 a
		Mean	104	1553.2	80.3 A	561.5 AB	2.37 A
ANOVA							
V					31.8 **	9.3 **	19.2 **
N					5.4 **	12.7 **	96.2 **
K					7.1 **	15.4 **	4.7 *
V × N					ns	ns	8.0 **
V × K					ns	ns	ns
N × K					ns	ns	ns
V × N × K					ns	ns	ns

GD: growth duration, IR: total solar radiation, IPAR: intercepted photosynthetically active radiation, IP: light interception percentage. Within each column, means followed by the same letters are not significantly different according to LSD (0.05). ns, non-significant. ** Significant at *p* < 0.01. * Significant at *p* < 0.05.

**Table 2:** Radiation use efficiency (RUE) and its related parameters of two hybrid rice varieties at maturity under different nitrogen rates in 2021.

Variety	Nitrogen	Potassium	GD	IR	IP	IPAR	RUE
(V)	(N)	(K)	(d)	(MJ m^−2^)	(%)	(MJ m^−2^)	(g m^−2^)
YLY900	N_90_	K_120_	106	1822.9	87.2 a	714.9 b	1.95 a
		K_160_	106	1844.1	87.2 a	723.4 b	1.95 a
		K_210_	106	1864	88.7 a	744.1 a	1.95 a
		Mean	106	1843.7	87.7 B	727.5 B	1.95 B
	N_120_	K_120_	108	1844.1	85.8 a	711.8 b	2.04 c
		K_160_	108	1864.0	87.4 a	732.9 b	2.12 b
		K_210_	108	1878.8	87.7 a	741.0 a	2.17 a
		Mean	108	1862.3	86.9 B	728.6 B	2.11 A
	N_180_	K_120_	110	1864.0	92.0 a	771.7 a	1.92 b
		K_160_	110	1878.8	92.4 a	781.5 a	2.09 a
		K_210_	110	1896.6	92.6 a	790.4 a	2.13 a
		Mean	110	1879.8	92.4 A	781.2 A	2.05 AB
QYHZ	N_90_	K_120_	98	1655.0	85.2 a	634.6 b	2.26 b
		K_160_	98	1675.1	85.6 a	645.0 b	2.32 a
		K_210_	98	1694.9	88.0 a	671.0 a	2.33 a
		Mean	98	1675.0	86.3 C	650.2 C	2.30 C
	N_120_	K_120_	100	1675.1	88.4 a	666 b	2.46 b
		K_160_	100	1694.9	89.2 a	680.4 b	2.50 a
		K_210_	100	1715.1	90.7 a	699.6 a	2.50 a
		Mean	100	1695.0	89.4 B	682.0 B	2.49 B
	N_180_	K_120_	102	1694.9	91.6 a	698.8 a	2.58 b
		K_160_	102	1715.1	92.9 a	716.9 a	2.59 b
		K_210_	102	1721.2	93.3 a	722.5 a	2.73 a
		Mean	102	1710.4	92.6 A	712.7 A	2.63 A
ANOVA							
V					ns	221.9 **	467.3 **
N					37.8 **	64.6 **	41.3 **
K					3.7 *	14.6 **	8.4 **
V × N					4.3 *	4.5 *	13.4 **
V × K					ns	ns	ns
N × K					ns	ns	ns
V × N × K					ns	ns	ns

GD: growth duration, IR: total solar radiation, IPAR: intercepted photosynthetically active radiation, IP: light interception percentage. Within each column, means followed by the same letters are not significantly different according to LSD (0.05). ns, non-significant. ** Significant at *p* < 0.01. * Significant at *p* < 0.05.

**Table 3:** Radiation use efficiency (RUE) and its related parameters of two hybrid rice varieties at maturity under different nitrogen rates in 2022.

Variety	Nitrogen	Potassium	GD	IR	IP	IPAR	RUE
(V)	(N)	(K)	(d)	(MJ m^−2^)	(%)	(MJ m^−2^)	(g m^−2^)
YLY900	N_90_	K_120_	105	2074.2	87.0 a	811.8 a	1.72 a
		K_160_	105	2083.5	87.6 a	821.2 a	1.73 a
		K_210_	105	2100.9	88.0 a	832.1 a	1.75 a
		Mean	105	2086.2	87.5 B	821.7 C	1.73 B
	N_120_	K_120_	106	2083.5	88.6 a	830.8 b	1.76 b
		K_160_	106	2100.9	89.2 a	843.2 b	1.85 a
		K_210_	106	2115.7	90.9 a	865.1 a	1.85 a
		Mean	106	2100.0	89.6 B	846.4 B	1.82 A
	N_180_	K_120_	107	2100.9	91.6 a	865.6 a	1.71 c
		K_160_	107	2115.7	92.2 a	877.7 a	1.83 b
		K_210_	107	2124.0	92.5 a	883.7 a	1.90 a
		Mean	107	2113.5	92.1 A	875.6 A	1.81 A
QYHZ	N_90_	K_120_	97	1978.7	85.1 b	757.3 b	1.79 b
		K_160_	97	1997.4	85.8 b	770.8 b	1.82 b
		K_210_	97	1998.0	87.0 a	782.5 a	1.89 a
		Mean	97	1991.4	86.0 C	770.2 C	1.83 C
	N_120_	K_120_	98	1997.4	88.5 b	795.0 a	1.95 b
		K_160_	98	1998.0	89.1 b	801.3 a	2.02 a
		K_210_	98	1988.5	90.5 a	810.0 a	2.06 a
		Mean	98	1994.6	89.4 B	802.1 B	2.01 B
	N_180_	K_120_	100	1998.0	91.1 a	818.9 a	2.10 b
		K_160_	100	1988.5	92.2 a	824.5 a	2.12 b
		K_210_	100	1995.8	92.6 a	831.2 a	2.27 a
		Mean	100	1994.1	91.9 A	824.9 A	2.16 A
ANOVA							
V					ns	165.8 **	515.5 **
N					55.2 **	68.4 **	164.2 **
K					5.3 *	10.1 **	51.7 **
V × N					ns	ns	60.7 **
V × K					ns	ns	ns
N × K					ns	ns	5.6 **
V × N × K					ns	ns	ns

GD: growth duration, IR: total solar radiation, IPAR: intercepted photosynthetically active radiation, IP: light interception percentage. Within each column, means followed by the same letters are not significantly different according to LSD (0.05). ns, non-significant. ** Significant at *p* < 0.01. * Significant at *p* < 0.05.

**Table 4:** Analysis of variance (ANOVA) of the F-values of grain yield (GY), aboveground total dry weight (TDW), harvest index (HI), and RUE and its related parameters at maturity from 2020 to 2022.

ANOVA	GY	TDW	HI	IP	IPAR	RUE
Y	3435.8 **	1762.6 **	46.7 **	727.8 **	5045.1 **	490.1 **
V	718.3 **	119.0 **	94.0 **	7.1 **	347.8 **	338.4 **
N	1638.1 **	697.4 **	ns	78.8 **	133.6 **	214.6 **
K	255.7 **	148.9 **	13.5 **	14.5 **	37.5 **	30.7 **
Y × V	402.5 **	153.2 **	32.4 **	10.6 **	56.8 **	225.8 **
Y × N	19.3 **	2.7 *	9.2 **	9.0 **	13.6 **	6.7 **
Y × K	4.1 **	3.2 *	ns	ns	ns	ns
V × N	49.1 **	77.2 **	4.3 *	4.1 *	3.2 *	44.2 **
V × K	ns	ns	ns	ns	ns	ns
N × K	4.2 **	4.4 **	6.0 **	ns	ns	3.3 *
Y × V × N	11.8 **	2.7 *	ns	ns	ns	ns
Y × V × K	ns	ns	ns	ns	ns	ns
Y × N × K	2.9 **	ns	3.7 **	ns	ns	ns
V × N × K	3.0 *	2.8 *	ns	ns	ns	ns
Y × V × N × K	3.6 **	ns	3.0 **	ns	ns	ns

Y: year. ns, non-significant. ** Significant at *p* < 0.01. * Significant at *p* < 0.05.

**Table 5:** Regression coefficients, standard error (SE), significance, and adjusted R^2^ values from multiple regression analyses for correlations between grain yield (GY) and intercepted photosynthetically active radiation (IPAR), radiation use efficiency (RUE), and harvest index (HI) in each rice variety.

GY	IPAR			RUE			HI			Adj.R^2^
	Value	SE	*p*-Level	Value	SE	*p*-Level	Value	SE	*p*-Level	
QYHZ	4 × 10^−3^	4.07 × 10^−4^	***	3.57	0.16	***	2.71	3.36	ns	0.90
YLY900	1 × 10^−3^	5.99 × 10^−4^	***	4.15	0.34	***	2.93	2.21	ns	0.70

ns, non-significant. *** Significant at *p* < 0.001.

## Data Availability

All data supporting the conclusions of this manuscript are provided within the manuscript.
